# Immunity, virus evolution, and effectiveness of SARS-CoV-2 vaccines

**DOI:** 10.1590/1414-431X202010725

**Published:** 2021-03-15

**Authors:** J.E. Belizário

**Affiliations:** 1 Hospital das Clínicas, Faculdade de Medicina, Universidade de São Paulo, São Paulo, SP, Brasil

**Keywords:** SARS-CoV-2, COVID-19, Immunity, Virus evolution, Vaccines

## Abstract

Phylogenetic and pathogenesis studies of the severe acute respiratory syndrome-related coronaviruses (SARS-CoVs) strains have highlighted some specific mutations that could confer the RNA genome fitness advantages and immunological resistance for their rapid spread in the human population. The analyses of 30 kb RNA SARS-CoVs genome sequences, protein structures, and functions have provided us a perspective of how host-virus protein-protein complexes act to mediate virus infection. The open reading frame (ORF)1a and ORF1b translation yields 16 non-structural (nsp1-16) and 6 accessory proteins (p6, p7a, p8ab, p9b) with multiple functional domains. Viral proteins recruit over 300 host partners forming hetero-oligomeric complexes enabling the viral RNA synthesis, packing, and virion release. Many cellular host factors and the innate immune cells through pattern-recognition receptors and intracellular RNA sensor molecules act to inhibit virus entry and intracellular replication. However, non-structural ORF proteins hijack them and suppress interferon synthesis and its antiviral effects. Pro-inflammatory chemokines and cytokines storm leads to dysfunctional inflammation, lung injury, and several clinical symptoms in patients. During the global pandemic, COVID-19 patients were identified with non-synonymous substitution of G614D in the spike protein, indicating virus co-evolution in host cells. We review findings that suggest that host RNA editing and DNA repair systems, while carrying on recombination, mutation, and repair of viral RNA intermediates, may facilitate virus evolution. Understanding how the host cell RNA replication process may be driven by SARS-CoV-2 RNA genome fitness will help the testing of vaccines effectiveness to multiple independent mutated coronavirus strains that will emerge.

## Introduction

Metagenomic analysis of microbial communities have provided deep insights into the origin and functional dynamics of infectious diseases in the entire living world. Direct genetic analysis of DNA/RNA molecules can reveal potential loss (deletion) or gain (duplication) of genes of the vectors and host cells that predispose or prevent human diseases (1). Why? Mutation is a natural process for species evolution and adaptation to new ecological niches and host switching. We are continuously changing our continents and habitats introducing chemical hazards and toxic substances that can mutate and increase transmissibility and contagiousness of evolutionarily distant pathogens. Some earth environments and cultural and behavioral factors are more conducive to the spread of common parasites, bacteria, and viruses across Asia, Africa, Eastern Europe, and the Middle East (1). Overtime, the modern *Homo sapiens* has survived over 20 plagues, such as influenza virus pandemic (Spanish flu), smallpox, cholera, measles, tuberculosis, and recently the coronaviruses (2). From the human microbiome studies emerged over two million microbial strains and species (3). Antibiotic resistance strains, such as carbapenem-resistant strains belonging to the *Klebsiella pneumoniae* ST258, have infected one-third of the human population and remain under active evolutionary transmission (4). Penicillin, the first antibiotic, discovered in 1928, may be completely innocuous to most bacterial infections in the near future (5). Bacteria and viruses can pass on their recessive or resistance genes to progeny a thousand timescale faster than humans. This unparalleled adaptive mutability and evolutionarily acquired resistance among bacterial and virus strains seem to be beyond Darwinian principles of evolutionary adaptation (6). Have eukaryotic cells developed counteracting intrinsic mechanisms that might be helping microorganism evolution? How will the human immune system evolve from the severe acute respiratory syndrome coronavirus 2 (SARS)-CoV-2 pandemic? Could SARS-CoV-2 mutated strains develop the capacity to replicate and transmit from vaccinated hosts? I present here a conceptual framework for understanding the virus and human immunity co-evolution, and some approaches and challenges in the vaccine development for SARS-CoV-2.

## How do immune cells respond to microbial infection?

In 1883, Elie Metchnikoff described phagocytosis as a process of cellular engulfment of solid particles when a small citrus thorn was injected into starfish larva (7). In the 1890s, Emil Behring and Kitasato Shibasaburo described anti-toxin activity of horse blood serum for treatment of diphtheria - a disease described by Hippocrates. The diphtheria toxin is produced by the bacterium *Corynebacterium diphtheria* infected by a bacteria virus - a bacteriophage. In 1897, Paul Ehrlich, the first Nobel Prize winner immunologist, proposed the side-chain theory to explain the antitoxin effect, e.g. the antibody mechanism of action (7). In the 1950s, MacFarlane Burnet and Niels Jerne were the protagonists of the two models built to explain how an immune response develops. They created the self and non-self concept, in which self are constituents of the human body, not triggering an immune response. Non-self are foreign substances, such as pathogens or tissues and organs genetically distinct that would trigger an immune response. Their discoveries about the natural selection theory for antibody formation and diversity are still widely accepted (8,9). Based on these fundamental findings, we began to understand the evolution and complex interplay between myeloid and lymphoid cells and their cooperative roles in innate (non-specific defense) and adaptive (specific defense) immune responses.

The humoral - or adaptive immune - system emerged about 500 million year ago when a jewfish transposon inserted the recombination activating gene (RAG) into the lymphoid cell genome of a jawed vertebrate. RAG-1 and RAG-2 are enzymes that recognize and cleave recombination signal sequences (RSSs) in DNA gene sequence, thereby promoting a somatic recombination (10). With the power of DNA technology, the major questions relating to antibody formation and diversity were solved in the 1970s. A fundamental study by Sussumu Tonegawa, awarded with Nobel Prize of Medicine in 1987, showed how recombination and somatic hypermutation cause the rearrangement of variable-diversity-joining (V-D-J) regions of immunoglobulin (Ig) genes (11). The somatic rearrangement of genes into the germline genome generates a repertoire of B cell receptors (BCRs) or diversification of immunoglobulins that enable randomly finding new antigens. Next, it was demonstrated that the same combinatorial joining and the somatic V-D-J recombination mechanism operate for generation of T cell receptors (TCRs) in T lymphocytes. TCRs target an infinite range of antigens that T cells will have to face over their lifetimes. A peptide from a pathogen protein is recognized by unique a TCR molecule through class I and II molecules of the major histocompatibility complex (MHC). The clearest example of this structural feature of T-cell recognition was first demonstrated by Zinkernagel and Doherty (12), for which the authors were awarded the Nobel Prize in 1996. These fundamental knowledges forwarded the recent technological advances in the field of immunology that allow organ transplantation, antibody-mediated immunotherapies, and development of vaccines against many infectious diseases.

Our first microbial contact occurs at birth. The initial microbial colonization of the infant gut with a great diversity of bacteria, virus, fungi, and parasites is essential for the education of the immune system and host-microbe co-evolution (3). The human gut is the residence of a viral microbiome composed mainly by bacteriophages and RNA and DNA viruses (13). Bacteria use a dynamic system of the adaptive immune defense named CRISPR (clustered regularly interspersed palindromic repeats) in which they pack viral RNA or DNA segments. In this way, bacteria and archaea memorize the RNA/DNA structure of bacteriophages and recognize and kill them at their re-infection (14). This adaptive immune defense is similar to RNA interference pathway of eukaryotic cells (14). Bacteria use the quorum-sensing mechanism - releasing molecules like N-acyl-homoserine lactones or peptides - to directly or indirectly stop pathogenic species overgrowth and limit their crossing through host tissue barriers. How does immunity evolve in humans to fight the pathogens they host? Early in life, large lymphocytes (lymphoblast) migrate into the intestinal lamina propria and initiate a mucosal immune system (15). They exert important roles in the gut-associated lymphoid tissue (GALT) maturation, diversification, and differentiation into B cells - B1 and B2 populations - capable to produce secretory IgA (SIgA) and IgM antibodies and long-lived plasma cells. Within these sites, specialized epithelial cells, stroma cells, and native B cells inhibit virulence and overgrowth of commensal bacteria carrying pathogenicity through low-affinity, cross-reactive, and specie-specific IgA/IgM antibodies. B cell antibodies also provide mechanismsto suppress pathologic reactivity against self, known as mucosal tolerance. Numerous germinal centers (GC) are colonized with various subtypes of dendritic cells (DCs), CD4^+^ T cells, cytotoxic T cells (CTLs), and natural killer cells (NKs), which are able to discriminate self and non-self luminal antigens from infected cells. However, an overreaction of immune cells against self as well as non-self microbial antigens is suppressed by thymic (t) or peripheral (p) CD4^+^CD25^+^ immunoregulatory T cell (Treg) population (16). Tregs possess a TCR repertoire that recognizes self and non-self antigen, thus they play a central role in B and T cell immune tolerance (17).

How does the immune system recognize an incoming pathogenic virus and acquire an immunological memory? The antigen-presenting cells (APCs) or innate immune cells, such as macrophages and dendritic cells, display a variety of pattern-recognition receptors (PRRs), or sensors of danger, specialized in the recognition of the pathogen's components, which are referred to as PAMPs and DAMPs (pathogen associated molecular pattern, damage-associated molecular patterns, respectively) (18). PAMPs/DAMPs released by pathogens and host cells bind and activate a variety of PRR, specifically, toll-like receptor (TLR) and nod-like receptors (NLRs), which recruit and activate the inflammasomes (18,19). Afterwards, a proteolytic process by enzymes in the immunoproteosomes or lysosomes generates a peptide epitope. Dendritic cells and macrophages present viral peptides to T helper CD4^+^ cells through MHC Class II molecules, or to cytotoxic CD8^+^ T cells through MHC class I molecules. Once activated, these cells drive the synthesis of hundreds of cytokines and chemokines, which carry messages for growth, differentiation, or cell death. B cells can respond directly to virus antigens and become activated by them and also interact with CD4^+^ T cells. IgM is the first high-avidity antibody produced by B cells against a pathogen (3-5 days after infection); the high-affinity and neutralizing IgG classes are produced after 2 weeks. This transition (shift) from high-avidity IgM to high-affinity IgG occurs with the aid of mRNA editing enzyme APOBECs (apolipoprotein B mRNA editing enzyme, catalytic polypeptide-like), which are single-stranded polynucleotide cytosine deaminases (20). These enzymes cause somatic hypermutation, increasing the transcripts of immunoglobulin variants in B cells and thus antibody diversity, e.g., variable regions in the antibody protein, to randomly recognize new antigens (20).

The formation of long-lived immunological memory in T helper CD4^+^ cells, cytotoxic CD8^+^ T cells, and B plasma cells after a primary infection is only partially understood (21). Studies done in mice and humans have demonstrated that epigenetic, and not genetic mechanisms, are responsible for imprinting chemical signatures (memory) in DNA regions and histone proteins using diverse specialized enzymes. In this way, groups of genes and their promoters are methylated or demethylated in memory cells and turn on if recruited to combat a second infection (21,22). Bone marrow progenitor myeloid cells of NK cells, innate lymphoid cells (ILCs), monocytes, and macrophages can also develop innate immune memory by similar mechanisms (23). Trained innate immunity can be induced with classical immune stimulants such as bacterial LPS (lipopolysaccharide) and BCG (*Bacille Calmette-Guérin*) vaccine prepared with *Mycobacterium bovis* (23). How can we detect and isolate epigenetically long-lived memory cells for passive cell immunization? Would this cell therapy work better than vaccination? Recently, we have learned how immune checkpoint molecules specifically dampen TCR-mediated intracellular pathways. In 2018, the Nobel Prize of medicine was awarded to James Allison and Tasuku Honjo for their contribution to novel cancer immunotherapy based on the negative immune checkpoint blockage. Specific monoclonal antibodies to immune checkpoint proteins increase cytotoxic activity of exhausted T cells that recognize foreign antigens and neo-antigens in cancer patients (24). Why can’t we apply immune checkpoint antibodies to reinvigorate an effector and memory CD8^+^ T cells in the context of vaccination?

## Virus-induced replication and repair in host cells

Viruses live intracellularly for hours or days and occasionally can integrate into host DNA causing genome instability (25). RNA viruses, such as influenza virus, dengue virus, and coronavirus, due to the error-prone and low-efficient RNA polymerases, tend to have high mutation rates during replication (26). Errors can be corrected by proofreading RNA exonucleases (26). SARS-CoVs have indeed acquired an enzyme able to enhance the overall fidelity (27). Along virus replication and assembly, deficient virion particles accumulate in the cytosol and trigger host cell death programs by apoptosis, pyroptosis, necroptosis, and autophagy (28). Many DNA and RNA viruses counteract cell death by apoptosis through expression of viral homologs of Bcl-2 family of antiapoptotic proteins and inhibitors of caspases, such as cytokine response modifier A (CrmA) (29). How cell death mechanisms are manipulated by SARS-CoV-2 viral proteins is not known yet. Most viruses trigger a DNA damage response (DDR), an emergency signaling pathway mediated by kinase enzymes ATM (ataxia telangiectasia mutated), ATR (ataxia telangiectasia and Rad3 related), and DNA-PKcs (DNA-dependent protein kinase catalytic subunit) in order to avoid host cell genome instability (30). Viruses hijack or inactivate the host cell repair machinery proteins to replicate while the cell cycle stops at the S phase (30). DDR provides open breaks in DNA molecules facilitating the integration of provirus (viral transduction). Curiously, host genomic DNA released by dying cells during a virus lytic cycle acts as endogenous DAMPs, thus serving as a danger signal that can cause intense inflammation (31,32). The cytosine deaminases of APOBEC family and adenine deaminases (ADAR1 and ADAR2) are RNA-specific editing enzymes (33). The APOBEC A3 members control cellular resistance to retroviruses, in particular the human immunodeficiency virus (33,34). The APOBECs enzymes convert cytosine to uracil, creating normal RNA, but they can cause G to A and C to U changes that lead to defects in single-strand RNA and single-strand DNA. These defects trigger viral destruction (33,34). However, APOBEC cytidine deaminase and ADARs adenine editing events can create quasispecies viruses that incorporate new mutations in viral proteins (35). Thus, mutants carrying new versions of peptide epitopes on their antigenic proteins survive and invade new cells.

What is known and expected about SARS-CoV-2 coronavirus infection and APOBEC antiretroviral defense? Recent studies have confirmed APOBECs motifs in mutational signature of the coronavirus genome (36–38). This mutation process was observed to operate in the mutagenesis process of Rubella virus isolates (38). This powerful editing mechanism might exert the essential role in editing new beneficial variant genes to protect the human genome of retrovirus invasion. On the other hand, hypermutation mediated by cytoplasmic A3s APOBECs by introducing SNPs and potential mutation on SARS-CoV genomic RNA may be conferring advantage to viral adaptation and transmissibility (39). The organization of an accurately curated sequence database and molecular epidemiology studies of divergent human coronaviruses with distinct mutational signatures will help us to answer these questions.

## Virus-induced host cell immune response

**Figure 1:**
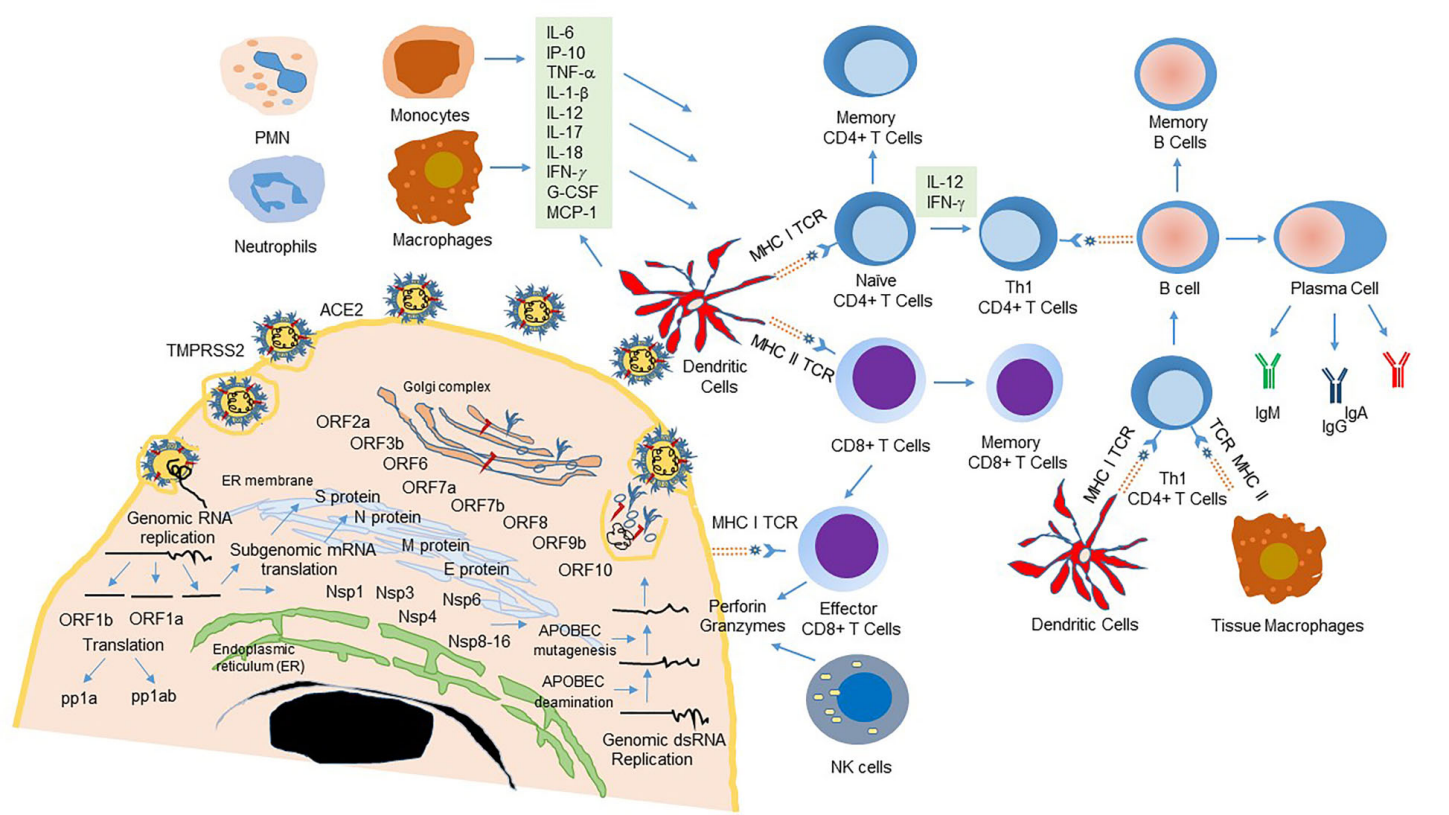
The severe acute respiratory syndrome-related coronavirus (SARS-CoV-2) and its life cycle and host immune response to viral infection. ACE2 is a cellular receptor in the lungs, arteries, heart, kidneys, and the intestine that binds to the viral (S) protein and is cleaved into S1 and S2 subunits by an extracellular protease. S2 is further cleaved and activated by TMPRSS2. The SARS-CoV is a large enveloped, single-stranded, positive-sense RNA virus (∼30 kb) in which 5' two-thirds encodes the two large open reading frames (ORFs) and is transcribed and translated into two polyproteins (pp1a and pp1ab) collectively termed the replicase. The 3' one-third of the SARS-CoV-2 genome encodes four essential structural proteins (S, spike, M, membrane, N, nucleocapsid, and E, envelope) and a set of functional non-structural (Nsp) and accessory proteins (ORFs), which are essential for evading immune response. SARS-CoV-2 is detected by various intercellular sensors, such as RIG I and TLR-3, -7/8, and -9. Viral peptides are presented via MHC I and II to tissue-residing APCs, such as dendritic cells and macrophages, which, in turn, can produce pro-inflammatory cytokines, including interleukin-6, (IL-6), IL-1, IL-17, TNF-α, etc. Cytokines modulate the adaptive immune response by recruiting and activating CD4+ T cells, CD8+ T cells, and B cells that orchestrate the production of antibodies IgM, IgA, IgG, and cytotoxic factors (perforin and granzymes) for killing the virus-infected cells. However, an unbalanced immune response can lead to hyper-inflammation and cytokine storm, causing ARDS and other clinical symptoms of severe COVID-19 patients. Cytoplasmic APOBECs by introducing cytosine to uracil changes and potential mutation of SARS-CoV genomic RNA may be conferring advantage to viral adaptation and transmissibility. ACE2: angiotensin-converting enzyme 2; TMPRSS2: type II transmembrane protease serine; MCP-1: monocyte chemoattractant protein-1; IP-10: IFN-*γ*-induced protein 10; TNF-α: tumor necrosis factor; MHC: major histocompatibility complex; TCR: T cell receptor; PMN: polymorphonuclear leukocytes; NK: natural killer; APC: antigen-presenting cells; TLR: toll-like receptor; RIG-I: retinoic-acid-inducible protein 1; ARDS: acute respiratory distress syndrome; ds-RNA: double‐stranded RNA; ORF: open reading frame. Partially adapted from Azkur et al. 2020 (40).

COVID-19 patients develop an acute respiratory distress syndrome, which can be mild, moderate, or severe, leading to high mortality of patients (2,40). Many factors and conditions can be predictive biomarkers of the virus-induced immune response and clinical outcomes, and only a few are known (2,40). Immunophenotyping studies have shown that SARS-CoV-2 virus infection induces a quite distinct antiviral program (41,42). The genomic RNA of SARS-CoV-2 contains 11 open reading frames (ORFs) that code to 16 nonstructural proteins, four structural proteins named spike (S), envelope (E), membrane (M), and nucleocapsid (N), and eight accessory proteins that interact with multiple cellular processes (43–45). The surface trimeric spike (S) protein binds to angiotensin-converting enzyme 2 (ACE2) receptor, and the complex is cleaved by cell surface protease TMPRSS2 to enter into the host cells (43). The innate immune response begins with the activation the PRRs intracellular signaling pathways, nuclear translocation of transcriptional activators NF-κB (nuclear factor - kappa B), and interferon regulatory factors (IRF3 and IRF7), and the production of interferons (type I) and interferon-stimulated genes (ISGs). The inflammasome activation and synthesis of the pro-inflammatory cytokines - interleukin-1, IL-6, IL-8, IL-12, tumor necrosis factor (TNF)-alpha, and types I and III interferons - trigger many signal pathways that enable CD4^+^T cells to polarize toward Th1 or Th2 immune response. However, SARS-CoV-2 infection leads to a dysregulation of the IFNs response; more specifically, ORF3b has been found to interfere with STAT (signal transducer and activator of transcription) nuclear translocation and IRF3 phosphorylation, resulting in the impairment of IFN-type signaling pathway (46). The interactions between virus proteins and cell surface receptors on neutrophils, macrophages, monocytes, endothelial cells, platelets, and lymphocytes cause strong activation and excessive blood clotting as well as intense damage in lung epithelial cells. These events are followed by the systemic cytokine storm and obstructive vascular process (47). The pro-coagulant effect is linked to expression of fibrinogen, SERPINs (serine protease inhibitors), factors II, III, and X, thromboxane, and TLR9 (48). Multiple organ dysfunction associated with sepsis-like trait in severe compromised patients is characterized by elevated plasma levels of IL-6, IL-7, TNF-α IP-10 (IFN-γ-induced protein 10), C reactive protein (CRP), and D-dimers, fibrin-degradation products that have been associated with disseminated intravascular coagulation (48,49). The levels of IL-6, TNF-α, and antiviral immunoglobulins are biomarkers for predicting severity and survival outcome of COVID-19 patients (49). Overall depletion of peripheral circulating leukocytes, including monocytes, dendritic cells, basophil cells, CD4^+^ T, and CD8^+^ T cells results in poor patient recovery and survival (50). COVID-19 patients develop virus-specific IgM, IgG, and IgA antibodies and T and B memory cells to elicit a robust T-cell or antibody response in SARS-CoV-2 infection (40). Antibody-mediated response to COVID-19 may be short-lived, according to a new longitudinal study that evaluated people who had the disease and recovered (50). Therefore, we do not know whether humoral or cellular responses are more relevant to a patient's recovery. The current challenge is to understand the complex innate and adaptive immune responses associated with diverse clinical manifestations induced by SARS-CoV-2 infection (41,42,50,51). These events are schematically represented on Figure 1.

## SARS-COV-2 vaccine design

The classical vaccination concept was created in 1796 by Edward Jenner who evaluated the efficacy of calf lymph or cowpox inoculation to protect children against smallpox disease (variola). New forms of vaccination and innovative technologies were developed for designing and producing vaccines on a large scale (52–54). Vaccines are typically composed of whole pathogens (e.g., poliovirus); some are live attenuated through culture passages, such as the Sabin live polio vaccine, or inactivated, such as the Salk inactivated polio vaccine, by exposure to chemicals or heat (referred to as toxoids). Vaccines prepared with purified antigens - one or various subunits of virus protein - elicit good humoral antibody response if administered with an exogenous adjuvant (55). Pathogen protein genes can also be inserted into a genetically-modified virus, for example, vaccinia, adeno-associated virus (AAV), poxviruses, and vesicular stomatitis virus, to produce recombinant viral-vectored vaccines or non‐pathogenic bacterial vectors, such as *Lactococcus* species. Modified Vaccinia Virus Ankara (MVA) is a replication-deficient viral vector that has been used to create promising novel multivalent vaccines for respiratory viruses (56). Virus proteins can be assembled as three-dimensional viral structures composed of virus-like particles (VLPs) with multiple target sites (54,57). Vaccine candidates are chosen through serological studies that determine the variability or conservation of the antigen epitopes (58). The structural analysis of amino acid epitopes on the influenza hemagglutinin (HA) and neuraminidase (NE) proteins showed that they display either highly variable or conserved cross-reactivity (58). The influenza virus subtypes with high genetic variability - named antigenic shift - usually develop resistance to adaptive immune responses and antiviral agents, which is why universal vaccines fail. However, studies undertaken during the 2009 H1N1 pandemic showed that the vaccine prepared with the head domain of the H1 hemagglutinin protein displayed limited variability and induced heterosubtypic immunity, e.g., cross-protection to emergent influenza virus subtypes (59). Epidemiological studies on the common cold coronaviruses OC43, 229E, and SARS-CoV-1 coronavirus strains have shown evidence of antigenic drift in the spike S protein (60). Phylogenetic studies on SARS-CoV-2 genome structure have demonstrated little genetic diversity and minimum structural difference to its relative SARS-CoV and MERS-CoV of *Betacoronavirus* from bats (61–63). However, several nonsynonymous mutations in ORF1b and ORF8, and unique point mutations in S-proteins were identified in virus isolates from different areas of China (44,62,64–66). A total of 74 sites were identified as potentially under positive selection along S1 and S2 domain in isolates from palm civets and humans (67). More interesting, it was found that SARS-CoV-2 variant carrying non-synonymous substitution D614G in the spike protein, which is localized in the TMPRSS2 cleavage site, has emerged with improved transmission across populations (68,69). Subtypes of SARS-CoV-2 carrying D614G mutation and three other linked mutations disseminated globally (63). Furthermore, it is predicted that SARS-CoV-2 future mutations through over ten thousand single nucleotide polymorphisms variants, mainly structural genes, may develop, and consequently, they will impact the effectiveness of candidate vaccines (44,63). Thus, the candidate vaccines may not be equally effective against all coronavirus strains.

All virus components can function as potential antigen; however, most viruses are first detected through their nucleic acids, specifically, dsRNA, ssRNA, and DNA molecules by host cell specialized cytosolic sensors and adaptors (19,29). The toll-like family, such as the TLR3, TLR7/8, and TLR9, as well as RIG-I (retinoic-acid-inducible protein 1), interferon-inducible protein 16 (IFI16), the interferon-inducible protein Z-DNA binding protein 1 (ZBP1), and melanoma-differentiation-associated gene 5 (MDA-5) are major sensors that recognize viral nucleic acids within the endosomes and autophagic bags through their helicase domain. The mitochondria-associated proteins-stimulator of IFN genes (STING), the mitochondrial tethering protein mitofusin 2 (MFN2), and the mitochondrial antiviral signaling protein (MAVS) are examples of adaptor proteins, which are anchored in the outer mitochondrial membrane where they interact with sensor proteins (19,29). Modified viral RNA and DNA and host mitochondrial DNA fragments act as a DAMP that contribute to self and non-self discrimination to activate immune response (32). COVID-19 patients develop antibodies to M, nsp6, ORF3a, N, and the spike (S) proteins (44,70). The S protein is composed of two domains: S1, the receptor-binding domain (RBD), and S2, the fusion domain. The neutralizing antibodies to the RBD of spike glycoprotein and nucleocapsid were found in high levels in serum of COVID-19 patients (50). Various strategies and regimes were applied to develop over 160 candidate vaccines to SARS-CoV-2, of which 30 potential candidates have advanced to II and III clinical phases, according to the World Health Organization, in August 2020 (Supplementary Table S1). The first inactivated whole SARS-CoV-1 virus vaccine demonstrated safety and high immunogenicity (71) as well as an inactivated whole SARS-CoV-2 candidate vaccine (72). The recombinant adenovirus vectored vaccine (73) and chimpanzee adenovirus-vectored vaccine (named ChAdOx1 nCoV-19) expressing the SARS-CoV-2 full spike protein (74) are ongoing randomized phase III clinical trials in various countries. The first lipid nanoparticle-encapsulated mRNA vaccine, called mRNA-1273, encoding the stabilized perfusion SARS-CoV-2 spike protein advanced to phase III (75). The second one, BNT162b1, a lipid nanoparticle-formulated, nucleoside-modified, mRNA vaccine that encodes SARS-CoV-2 spike glycoprotein RBD linked to T4 fibritin-derived foldon trimerization domain - to increase its immunogenicity - advanced to phases I and II (76). The self-replicating noninfectious engineered RNA vaccines that express virus-like particles do not assemble to form a virus *in vivo*. A clinically relevant concern with all vaccines is induction of inflammatory response and cross-reactive antibodies that could cause antibody-dependent enhancement (ADE) side effects, as documented for dengue and respiratory syncytial virus vaccines in humans (77). To determine whether a vaccine will be successful in the late stages (phase III) of development, we need to define dominant epitopes to helper CD4^+^ T-lymphocytes, cytotoxic T-lymphocytes, and B-cell receptors as well as peptide-MHC complexes in vaccinated people compared to recovered COVID-19 patients. The repertoire of the HLA molecules - a haplotype - may be associated with the susceptibility to SARS-CoV-2 in different people. The comparative mapping of immunogenicity will help to choose the best epitopes that are recognized by a larger number of HLA alleles in inter-populations with genetic differences. Could this information be transduced into the development of an optimal SARS-CoV-2 vaccine? Reverse genetic systems for coronaviruses are now available for generation of infectious clones and larger panels of derivative mutants (78). It is expected that the use of such biotechnological approaches will facilitate the pathogenesis studies of wild type and mutant types containing gain and loss of function mutation in cell lines and animal models.

Most viruses are mucosa-transmitted; thus, understanding the rules that mediate immunity at mucosal tissues remain a critical issue (79,80). Oral and intranasal vaccines generate the systemic humoral and cytotoxic T cell responses, and most importantly, the secretion of sIgA by plasma cells in local and regional MALT, GALT, nasal-associated lymphoid tissue (NALTs), and the airway bronchus-associated lymphoid tissue (BALT) (15). One study in mice indicated that intranasal mucosal immunization with SARS-CoV VLPs assembled in a recombinant baculovirus (rBV) induced sIgA and IgG against SARS-CoV-1 (81). Another study demonstrated that mice with intranasal immunization with ChAd-SARS-CoV-2 developed mucosal IgA and T cell responses (82). SIgA and systemic IgA antibodies act by blockading epithelial receptors and thus inhibiting the entrance of microbial and viral pathogens within the specialized mucosal epithelium and mucosal lymphoid follicles. Therefore, mucosal IgA can mediate immune exclusion as well as induction of oral tolerance in some cases. This paradox illustrates the complexity of the mucosal immune system. Innate lymphoid cell (ILC) subsets within mucosal tissues play important roles in the innate and adaptive mucosal immunity (83). ILCs do not express TCR as CD4^+^ T cells and CD8^+^ T cells, but they can modulate their acquired immune response through production of cytokines that provide the local Th1/Th2 balance (83). ILC-1 subtype predominantly induces Th1 response known to be more specific to intracellular pathogens (virus), while ILC-2 subtype provides Th2 immune response to fight large parasites (helminths) and extracellular microbes. Pulmonary ILC-2 subtype is implicated in chronic respiratory inflammation and diseases (84). Studies have shown that Th1 is predominant in responses in convalescing COVID-19 cases (50). ILCs express MHC II molecules capable of presenting viral antigens to helper CD4^+^ T and modulating a Th1 response after mucosal vaccination. Adjuvants are immunostimulants added to vaccine formulations aiming to activate local immunocompetent innate immune cells to release cytokines (IL-12 and type I IFN-α) and priming CD4^+^ T helper cells to become Th1- or Th2-immune polarized (55,85). Aluminum-based mineral salts (Alum) have been used as adjuvant for nearly a century (86). Alum is an inducer of Th2 immune response that is considered detrimental to COVID-19 patients (41,47). From lessons learned with bacteria-contaminated vaccines, in particular polysaccharide LPS - a TLR4 agonist - new chemical classes of adjuvants with high reactogenicity have been identified, such as monophosphoryl lipid A (MPL), squalene (oil emulsion), CpG-containing oligonucleotides (CpG), flagellins, imiquimods, and bis-(3′,5′)-cyclic dimeric guanosine monophosphate (c-di-GMP) (86,87). These compounds are classified based on the mode of action, the agonist binding specificity to innate immune receptors, and activation of conventional and plasmacytoid DCs (85). CpG can enhance mucosal immune responses (81). For further clinical development of safe mucosal vaccines for SARS-CoV-2, we need to learn how to control immunogenicity properties of newly developed adjuvants to avoid tolerance commonly observed after mucosal vaccination (85–87).

## Concluding remarks

The mammalian immune system is under evolutionary pressure in the battle to survive with continuous spread of bacterial, fungal, parasitic, and viral pathogenic zoonotic diseases. So far there are no clear biomarkers that help differentiate SARS-CoV-2 infectiousness and outcomes in asymptomatic to mild, moderate, and severe cases of COVID-19. Studies suggest that novel progeny from the SARS-CoVs in different species have accumulated existing mutations giving them advantages. The transition has favored beneficial traits to transmit and co-evolve in human host cells in the course of the global spread. We need to learn how to control selection pressures on the dynamic immune response and RNA editing processes that have allowed viral mRNA mutation, recombination, and genetic stability to stop the new viral strains to evolve. Seromic-based screening in cured COVID-19 patients will be useful to identify new protein variants that escape strain-specific adaptive immune responses. Certain peptide epitopes and HLA alleles, which are increasing or decreasing, could help to identify susceptibility to COVID-19 and host response to the infection. Together, these results will contribute greatly to screening potential protein epitopes and innate immune evasion factors in datasets. Ideally, this information will support innovative strategies for antiviral therapies, new diagnostic tests of T cell immunity, and peptide vaccine candidates. In addition, epidemiology and genetic data should inform biomarkers for vaccine success and failure. How can epigenetic biomarkers of adaptive and innate immunity memory cells be explored to increase SARS-CoV-2 vaccine effectiveness? Could mucosal adjuvants that induce cytokine burst predominantly from ILCs provide a better response to SARS-CoV-2 vaccine? These are some scientific questions that are worth pursuing in the future. We hope scientists will come up with new ideas and solutions in different forms of serendipity to answer these questions before a novel virus pandemic happens.

## Supplementary Material

Click here to view [pdf].
